# The rapid growth in Mendelian randomization studies

**DOI:** 10.1007/s10654-025-01317-7

**Published:** 2025-11-06

**Authors:** Gibran Hemani, Stefan Stender, Frank J. Wolters, Albert Hofman, George Davey Smith

**Affiliations:** 1 Medical Research Council Integrative Epidemiology Unit, Department of Population Health Sciences, Bristol Medical School, University of Bristol, Bristol, BS8 2BN UK; 2 Department of Clinical Biochemistry, Rigshospitalet, Copenhagen University Hospital, Copenhagen, Denmark; 3 Department of Epidemiology, Erasmus MC University Medical Center, Rotterdam, The Netherlands; 4 Department of Epidemiology, Harvard T.H. Chan School of Public Health, Boston, USA

Mendelian randomization (MR) has shown utility in effective causal inference across different branches of epidemiology [1, 2], and it is difficult to ignore the rapid rise in numbers of papers using MR now appearing. Reviewer requests for Mendelian randomization studies, including from the European Journal of Epidemiology (EJE), are gracing our inboxes at an unprecedented rate, and there is a growing ubiquity of MR studies across the epidemiological literature [3]. As of mid 2025, a PubMed search for ‘Mendelian randomization’ in the title or abstract returns around 16,000 results since its first occurrence in 2003. Manual inspection of the hundred or so most recent MR papers in the literature indicates that the large majority of these papers show signs of being low quality, often reporting on relationships of variables that have little justification to be analysed [4], failing to discuss the gene-environment equivalence principle to justify the study design [1], failure to use STROBE-MR reporting guidelines [5, 6], and often with clear methodological errors [7, 8].

To what extent should we be concerned? One perspective is that when a set of analytical tools and approaches mature, they become available to contribute to the understanding of a wide range of epidemiological questions. Another perspective is to recognise that boom and bust cycles have happened many times before with other methodological techniques. Systematic reviews and meta-analysis experienced a similar massive influx of low quality studies [9], yet high quality systematic reviews remain an impactful and critically important tool [10]. However, the problem of differentiation between useful, high quality, research against the vast backdrop of poor and redundant publications is a major issue, recognised by researchers and funding agencies alike [11].

There is clearly a risk that a deluge of low-quality MR papers will lead to reduced credibility for all MR studies. Well-designed and carefully executed MR studies are a vital component of epidemiological research, and if they lose credibility, subsequent efforts to triangulate evidence on important causal questions will be impoverished. Hence commentators have lamented the misuse of MR and called for stricter guidelines on how MR is performed and papers are reviewed and selected for publication [3, 5, 12–14]. These will certainly be beneficial, but we argue that (as is generally true for successful interventions) in order to remedy this problem, we must understand its cause.

## The factors driving prolific output of MR studies

**Fig. 1:**
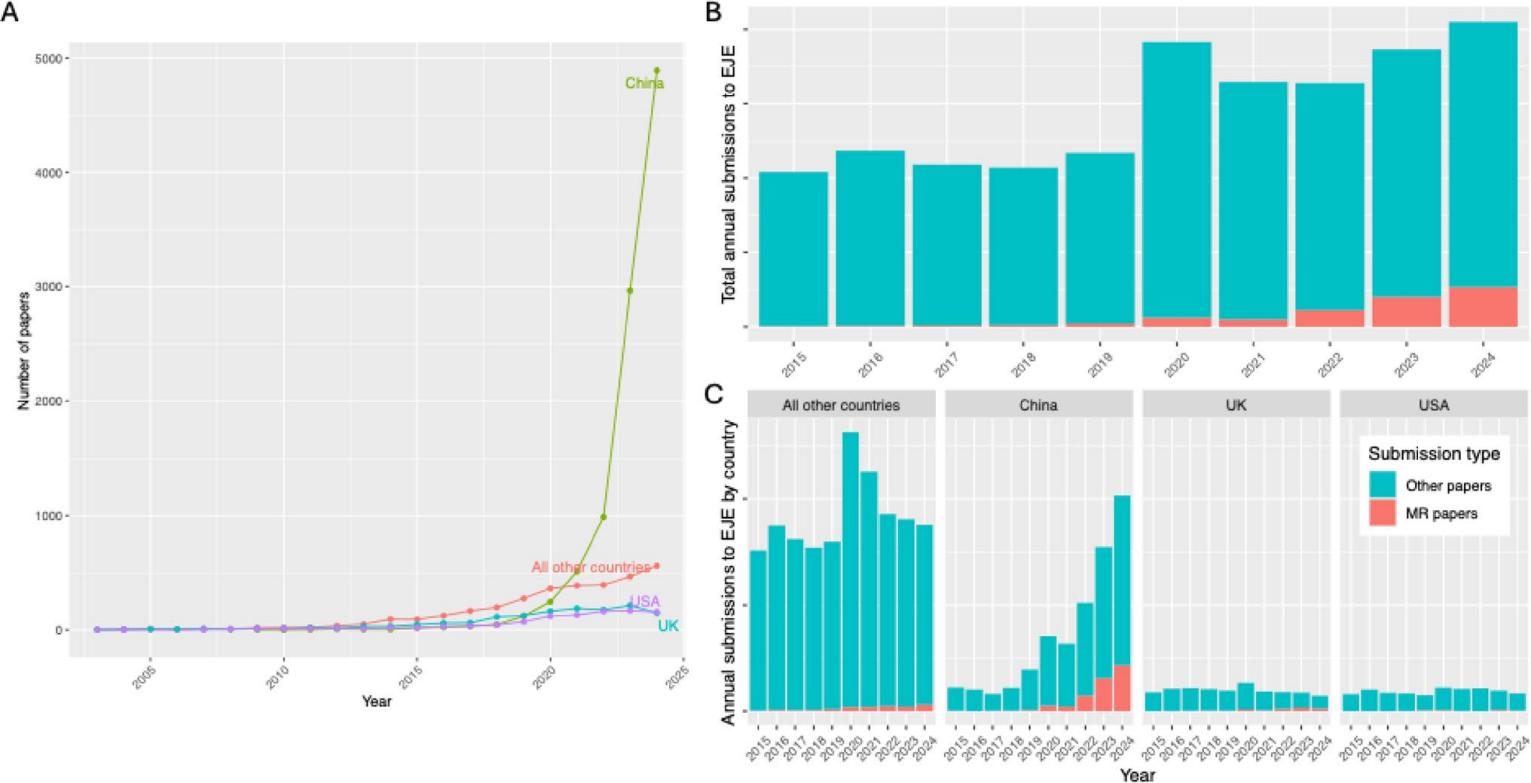
**A** The number of papers published per year according to PubMed based on the search term ‘Mendelian randomi[z/s]ation [tiab]’. Papers are stratified crudely based on the country of the primary affiliation of the first author, and as we discuss in the main text it is important to understand the nuances around these patterns. Only the top three contributing countries are labelled. **B** Total papers submitted to EJE stratified by type of paper. The spike in 2020 represents an influx of submissions during the COVID pandemic. Absolute numbers not shown on the y-axis per publisher request. (**C**) As in (**B**) but also stratified by country of the primary affiliation of the first author. Though the growth in the number of MR papers submitted to the EJE is largely driven by authors affiliated with institutions in China, these papers represent a minority of the growth in papers from China, and is likely partly a passenger of the general growth in scientific activity from this demographic. Submission counts here are absolute and do not account for population sizes or the number of researchers by demographic grouping

We performed a systematic analysis of the MR papers in PubMed, to quantify the growth in MR literature and identify its drivers. Similar to EJE submissions, we found in PubMed that the number of MR papers grew exponentially, and that this growth is driven by authors primarily affiliated to institutions in China [12] (Fig. 1**a**). The total number of papers submitted to EJE has risen from 1040 submissions in 2015, to 2049 submissions in 2024, and the fraction that is due to MR papers has also grown (Fig. 1**b**). Overall, the annual percentage of EJE submissions represented by MR papers grew from 3.1% in 2020 to 13.0% in 2024. This percentage was broadly similar between countries (e.g., for UK: 6.0 to 17.8%), albeit slightly higher among papers from China (7.9 to 21.5%).

Striking patterns of growth in scientific output from authors affiliated with institutions in China can be seen when expanding the view beyond the narrow lens of MR studies. Figure 1**c** shows that while the growth in MR papers submitted to the EJE far exceeds that from other countries, so too does the growth in all other types of submissions, and that the MR papers are a relatively modest fraction of those overall scientific outputs. This reflects a wider trend that not only does China lead in terms of volume of global scientific output [15], but as of 2024 it also leads in terms of the share of the highest quality of research outputs [16, 17]. This historic feat has relevance to the discussion about decolonisation of scientific research, indicating that scientific output need not be centred within the so-called global North [18]. The recent growth in China’s overall research output certainly has been rapid (estimated doubling time since 2015 of non-MR paper submissions to EJE is every 2.7 years, SE = 0.31), yet its production of MR papers has grown faster still (estimated doubling time since 2015 of submissions to EJE is every 1.2 years, SE = 0.13).

We suggest that within any research context there are likely two overlapping ecosystems of research activity, which may partially explain the MR paper publishing patterns in China. One epitomises high quality research. Major contributions have been made to MR methodology driven by researchers affiliated to Chinese institutions [19–21], major cohort studies are being developed [22, 23], and careful and diligent applied research is also routinely performed [24]. In parallel, several studies have documented particularly strong incentives to publish in China [25], with those at many levels in academic and healthcare institutions being expected to publish in international journals [26–28]. Whilst a pressure to publish is observed in academia globally, initiatives like DORA are redefining academic best practice, moving away from bibliometrics as the only measure for academic recognition and reward. As of yet, the DORA declaration has been signed by organisations in 166 countries, but uptake differs between countries, including for example 183 organisations in the U.S. and 289 in the U.K., compared to 9 in China [29]. Anecdotal reports through personal communication indicate a lack of high quality training materials available to meet demand, and social media enabling low quality training materials to proliferate. Laudably, some efforts are being made for training materials and opportunities to become available for a growing diversity of scholars [30], though more must be done.

**Fig. 2:**
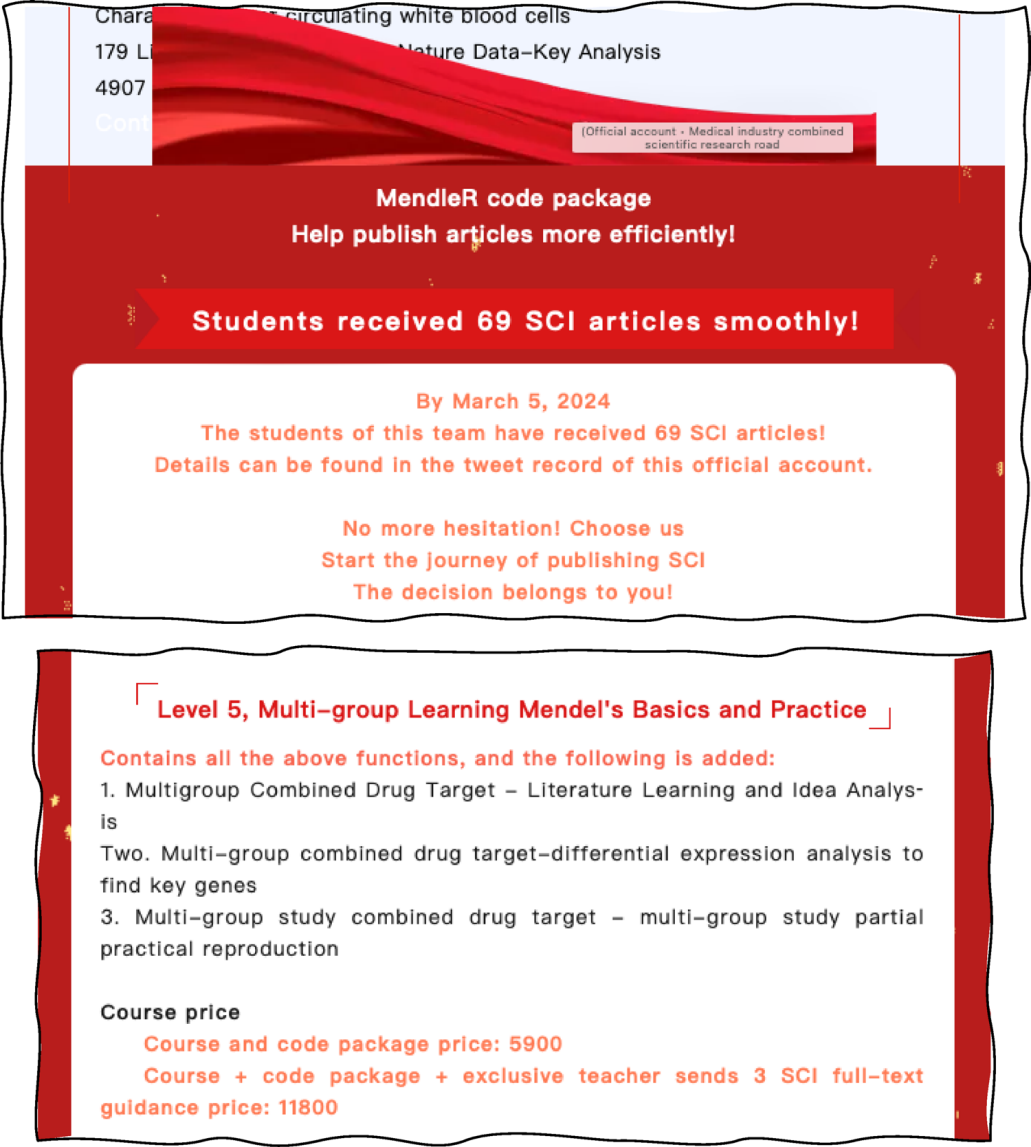
A screenshot from a company offering services to produce MR papers [screenshot taken 2024/10/18, with automatic translation from Chinese Mandarin to English]

Contributing to this high volume publishing is that much of the analytical software required for MR analysis has been linked to large-scale GWAS databases, enabling automation of basic MR analyses [31–34]. These resources are free to use and open source, and have been co-opted by research paper mills: opportunistic companies that offer quick and low effort solutions to those struggling to meet publication expectations (Fig. 2), at a financial cost to the clients [35], a credibility cost to the discipline, and arguably to science in general [36]. While research paper mills are a global phenomenon [37, 38], bibliometric analyses indicate that the vast majority of papers retracted due to use of paper mills are from institutions based in China [39]. China's supreme court has recently renewed attempts to crack down on paper mills [40, 41].

It is challenging to separate output from China into these two ecosystems. But the first ecosystem that engenders responsible and diligent scientific publishing would most likely follow the modest growth trends seen in the rest of the world. The second ecosystem that is driving exponential growth, amplified by structural pressures to publish, may lead to unintended adverse consequences. For example the many excellent China-based researchers making valid contributions to the epidemiological literature are perhaps the biggest victims of paper mills, because their research may be dismissed out of hand due to lazy stereotyping and generalisations.

## The role of automated tools

In 2016 it was noted that MR based on GWAS summary data was on the rise, at that point outstripping the growth of individual-level MR analyses [42]. Notably, in that paper it was discussed that some studies were harmonising datasets incorrectly, leading to invalid results. Today, the automation of the analysis using open source and widely scrutinised analytical software means that simple analytical errors are likely substantially reduced. The MR toolkit also becomes more accessible to a much wider diversity of researchers, including those from more under-resourced research environments, or researchers whose core skillset is not in epidemiological methods.

The OpenGWAS / MR-Base platform can automate many MR analyses that are based on GWAS summary data. It has seen a massive increase in users over the past few years, going from around 20k annual users in 2020 to 150k annual visitors this year. Approximately 90% of the increase is from users located in China, and likely contributes to the overall exponential growth in MR journal submissions and publications. There is also a massive increase in database searches per user, that implies that p-hacking might be being employed to drive positive results [43], which will give rise to publication bias. At the same time, such automated tools make it trivial to conduct analyses that are scientifically nonsensical, yet appear to be technically valid [44].

The world of paper mills is not well understood in the broader scientific community [36], and we don’t have a gauge on the scale of their contributions to the MR literature. That large numbers of recent MR publications appear relatively formulaic [14] could be indicative of paper mill activity, but it’s also possible that these are works produced by scholars new to the field, which has an entirely different set of implications for the type of response required.

Hence the contribution of these automated platforms to epidemiology is double-edged in nature. They can accelerate valid contributions to epidemiological research with diligent use, and can provide an accessible gateway into performing research for emerging scientists. Yet they can also invite poor scientific practice through uninformed application. They are also easily exploitable by non-conscientious enterprises such as the research paper mills. Automation of analytical tools is now being integrated with large language artificial intelligence models, facilitating entire papers to be produced with publicly available data and minimal discipline-related expertise [45, 46]. It may be difficult to discern at the submission stage between a manuscript that is the product of AI being used by an individual or a research paper mill, especially as detection due to data fabrication [47] becomes less relevant in the MR context due to the sheer abundance of real data. Understanding how we manage issues arising from research paper mills is one aspect of what is the now very real prospect of research being driven and produced by artificial intelligence.

## A perspective on diversity

In discussing this topic we must acknowledge an important power imbalance between those who hold positions of authority in any field, e.g. through editorial and peer reviewing roles that may have arisen through merit, unearned privilege or both, versus individuals from historically lower-opportunity demographics. There is a risk of those in power acting as unfair gatekeepers. We advocate that the scientific literature in general is strengthened by including a wider diversity of scholars. It is important to conceptually separate multiple trends that may be occurring simultaneously, and be cautious not to couple per se the growing diversity with the fraudulent practices through paper mills.

**Fig. 3:**
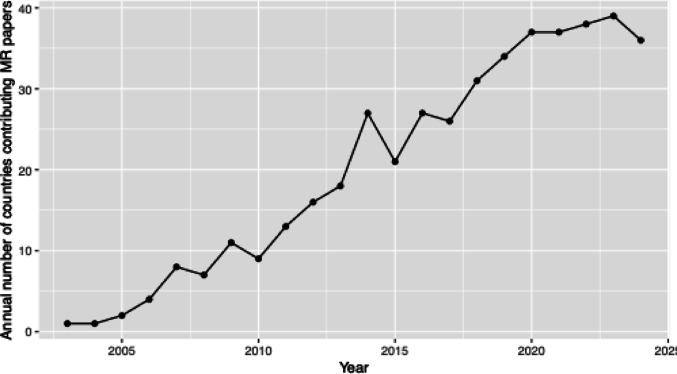
Number of countries represented by primary affiliation contributing at least one MR paper for a given year

The international growth of MR is much broader than what has been discussed so far (Fig. 3), with 62 countries contributing main author papers to the MR literature to date, compared to 45 countries in 2020. This diversity is likely at least in part enabled by the public availability of GWAS summary statistics, which although currently predominantly representing samples clustering by European ancestry [48], can be informative for causal inference across global contexts [49]. However, in order to meaningfully build a global community for MR we must ensure that analytical tools are appropriate to be applied to diverse ancestral groups, and that extensive investment is being made to redress the imbalance in global representation of biomedical data. A more productive vision of a rapidly growing MR literature is one in which the epidemiological questions that have previously been neglected due to an absence of domain expertise and data start to be addressed. Much of the responsibility in realising this vision lies at the feet of those who currently do hold positions of power and authority in the field, by building equitable partnerships and making wise investment decisions.

## Implications for biomedical journals

The growing ecosystem of methods and data for MR means that analyses that were once groundbreaking [50] can now be performed trivially in a matter of minutes, and perhaps even to a better standard. Such developments are a hallmark of progress [51]. However, while many aspects of performing MR have become *technically trivial*, in parallel we are becoming increasingly aware that the limitations of these methods are *scientifically non-trivial* and demand rigorous justification and supporting analysis [1, 3, 52–54]. The limitations for one research question are likely distinct from the limitations in another, so a one-size-fits-all approach to MR is unlikely to be valid. Below we discuss some broad approaches to examining the quality of an MR paper submitted for peer review.

**Reporting guidelines** The EJE will not send for review any MR papers that are submitted without the STROBE-MR guidelines having been completed [5, 6]. Whilst this too can be gamed, if the completed checklist is actually checked against the paper then serious problems will often be revealed. We emphasise that all code should be made available at the submission stage for review.

**Gene-environment equivalence** STROBE-MR states authors should “discuss whether the gene-environment equivalence assumption is reasonable”^6^. This is the fundamental MR principle [1, 55], and when interrogated rapidly exposes those papers that haven’t been designed to address the scientific validity of the use of MR for the research question.

**Emerging methods** There are threats to MR beyond the avalanche of summary-based MR studies generally published in low status journals. New methods continue to emerge, aiming to both improve the reliability of MR and expand the use cases for which it can be applied. Scientific methods typically follow a developmental cycle: initial proposal and evaluation, wider application and continued evaluation, on-going refinement and potentially eventual replacement. Here the role of journals is to ensure that the strength of any empirical claim is commensurate with the degree of evaluation that the methodology has thus far undergone by the broader scientific community. An example of how the field has failed in this regard has emerged recently, where empirical analyses using non-linear MR methods were published in high status journals [56, 57] and incorporated into clinical guidelines [58], only for subsequent examination to reveal important flaws in these methods [59–61], with the subsequent retraction of the original papers.

**New data resources** A large fraction of possible MR analyses using existing GWAS summary data have been generated and made available on resources like epigraphDB [62, 63] and OpenTargets [64]. Therefore, if the only analysis being presented is a summary-level MR analysis, using existing software and analytical methods, and using GWAS summary data that have already been published, the paper is likely offering nothing over and above what is already readily accessible online. However it certainly is possible to synthesise results from existing data resources into an empirical study that distills new epidemiological insights [65]. Such studies tend to (a) acknowledge that they are based on pre-existing summary data, (b) be well-motivated in their design explaining their novelty, and (c) combine several strands of genetic evidence to support any conclusions about causal inference.

**Triangulation** Our hope is that MR will be included as part of triangulation of evidence exercises [66, 67], either triangulating within genetic epidemiological study designs, or by combining genetic evidence with other independent forms of evidence [40]. However a concern arises even here—a rapid increase in papers has emerged which combine observational analyses of the NHANES study with a summary-based MR analysis of the same— sometimes meaningless—question [46]. Hence, the triangulation exercise must be well motivated and justified.

The threats and opportunities for valid causal inference in the epidemiological literature are continually developing, and criteria for publication will need to respond accordingly. In summary, the EJE considers MR a valid approach when applied judiciously, and particularly within a triangulation of evidence framework, but stringent screening will be applied in order to exclude inadmissible papers from the review process.

## Data Availability

Data and code for analyses are available here: https://github.com/mrcieu/mr-pubmed-abstracts.
